# Multicomponent reactions in nucleoside chemistry

**DOI:** 10.3762/bjoc.10.179

**Published:** 2014-07-29

**Authors:** Mariola Koszytkowska-Stawińska, Włodzimierz Buchowicz

**Affiliations:** 1 Faculty of Chemistry, Warsaw University of Technology, ul. Noakowskiego 3, 00-664 Warszawa, Poland

**Keywords:** multicomponent reaction, nucleoside analog, nucleoside antibiotics, nucleoside construction, nucleoside modification

## Abstract

This review covers sixty original publications dealing with the application of multicomponent reactions (MCRs) in the synthesis of novel nucleoside analogs. The reported approaches were employed for modifications of the parent nucleoside core or for de novo construction of a nucleoside scaffold from non-nucleoside substrates. The cited references are grouped according to the usually recognized types of the MCRs. Biochemical properties of the novel nucleoside analogs are also presented (if provided by the authors).

## Introduction

**Figure 1:**
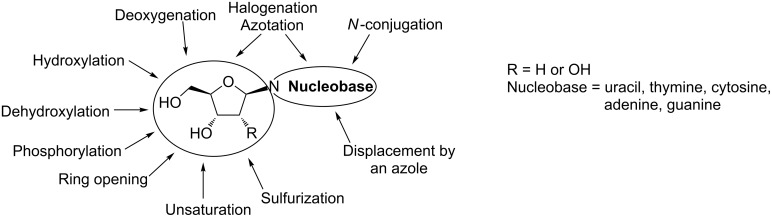
Selected chemical modifications of natural ribose or 2'-deoxyribose nucleosides leading to the development of medicinally important nucleoside analogs.

Chemical modifications of natural ribose or 2'-deoxyribose nucleosides resulted in the development of a group of compounds referred to as nucleoside analogs (Figure 1). The essential role of nucleoside analogs in medicine is reflected by the fact that currently thirty-six compounds from this class are used throughout the world in the therapy of viral or cancer diseases [1]. Moreover, several novel nucleoside analogs (including those embedded in versatile conjugate or pronucleotide scaffolds) are under clinical or preclinical trials [1]. Recent studies have also revealed a potential of nucleoside analogs as radiopharmaceuticals [2–6], antibiotics [7–9], anti-infective agents [10–12], or molecular probes [13–14]. Taking into account the importance of nucleoside analogs in medicine and biotechnology, there is a considerable interest in the development of simple and efficient synthesis of these compounds.

Multicomponent reactions (MCRs) represent an excellent tool for the generation of libraries of small-molecule compounds, for instance they are indispensable for the structure–activity relationship (SAR) studies. Many excellent comprehensive reviews on MCRs have been published. The reviews have covered the significant topics in this field, such as: (a) the applications of MCRs in the drug discovery process [15–20], or in the total synthesis [21–22]; (b) strategies developed for the construction of new structural frameworks [23]; (c) the use of specific building blocks [24–28], reagents [29–32], catalysts [33], reaction conditions [34–35], or preparative techniques [36] in MCRs; (d) methods for the design of new MCRs [37–38]; or (e) higher-order MCRs [39]. However to date, the application of MCRs in the chemistry of nucleoside analogs has not been methodically discussed. To the best of our knowledge, the only review articles in this field were published from the Dondoni research group [40–41] or from the Torrence research group [42–43], and they were limited to the results obtained by these groups.

The present review covers reports published up to October 2013, and is devoted to the employment of MCRs in the synthesis of nucleoside analogs. The references were selected in accordance with the definition of a MCR given by Ugi et al.: “a multicomponent reaction comprises reactions with more than two starting materials participating in the reaction and, at the same time, the atoms of these educts contribute the majority of the novel skeleton of the product” [44]. In this review, we understand educts as compounds that contribute carbon atoms to the MCR product [45]. By the analogy to nucleosides included in the DNA/RNA nucleic acids, this review is limited to MCRs involving furanosyl nucleosides as (i) reaction components, or (ii) products obtained from non-nucleoside substrates. The cited references are grouped according to the usually recognized types of the MCRs [46].

## Review

### 1. The Mannich reaction

**Scheme 1:**
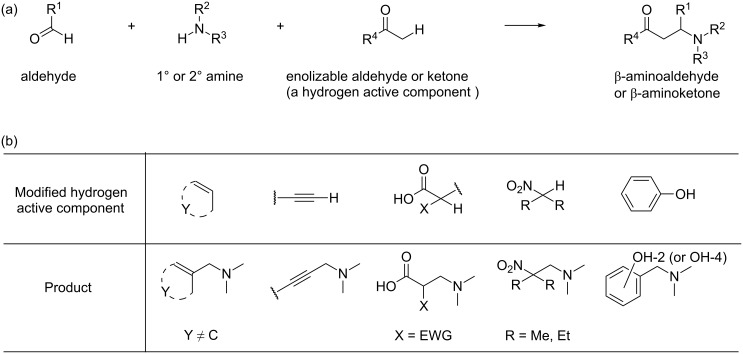
(a) Classical Mannich reaction; (b) general structures of selected hydrogen active components and structures of the resulting Mannich reaction products.

The classical Mannich reaction yields β-aminoaldehydes or β-aminoketones and involves: an aldehyde, a primary (or a secondary) amine, and an enolizable aldehyde (or ketone) (Scheme 1a) [47–48]. The use of a hydrogen active component other than an enolizable aldehyde or ketone leads to a variety of structurally diverse products (Scheme 1b). The Mannich reaction products (commonly named as Mannich bases) can serve as starting materials in the syntheses of a variety of compounds.

**Scheme 2:**
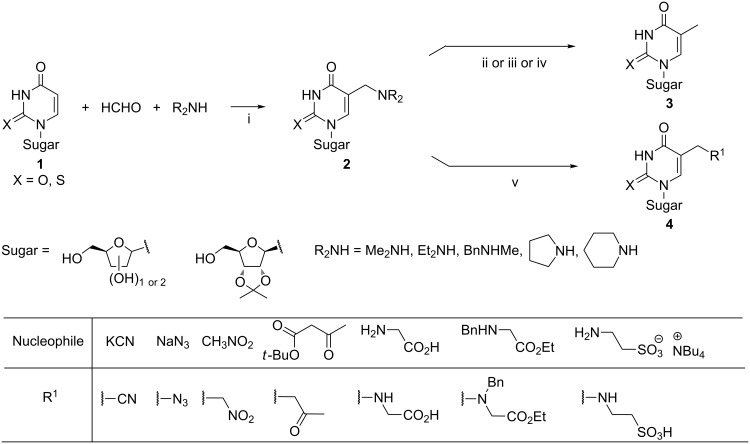
Reagents and reaction conditions: i. H_2_O or H_2_O/EtOH, 60–100 °C, 7 h–10 d; ii. H_2_, Pd/C or PtO_2_; iii. (1) 4-methylbenzenethiol, (2) Ni-Ra; iv. (1) MeI, (2) NaBH_4_; v. (1) MeI, (2) nucleophile.

The employment of a nucleoside as the hydrogen active component has been one of the most common variants of the Mannich reaction. Treatment of uracil (or 2-thiouracil) nucleosides **1** with aq formaldehyde and a secondary amine (i.e., dimethylamine [49–50], diethylamine [51–52], *N*-methylbenzylamine [49], pyrrolidine [53–54], or piperidine [55–56]) at temperatures ranging from 60 °C to 100 °C afforded the corresponding 5-(alkylaminomethyl)pyrimidine nucleosides **2** (Scheme 2). Compounds **2** served as precursors to a variety of compounds. The transformations leading to thymidine or its derivatives **3** involved: (a) the metal-catalyzed hydrogenolysis of products **2** [51–52,54–55] (or their 5-(4-tolylthio)methyl derivatives [57]), or (b) the reduction of methylammonium iodides derived from compounds **2** with sodium borohydride [53]. Compounds **4** were achieved by treatment of the corresponding methylammonium iodides with an organic nucleophile [56,58–60]. As studies on the synthesis of 5-taurinomethyluridine showed [60], this two-step procedure was much more efficient than a direct Mannich reaction involving taurine, formaldehyde and 2',3'-*O*-isopropylideneuridine [61].

**Scheme 3:**
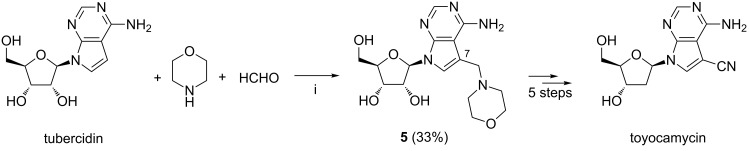
Reagents and reaction conditions: i. H_2_O, 90 °C, overnight.

Watanabe et al. described the synthesis of 7-(morpholinomethyl)tubercidin **5** by heating tubercidin, 37% aq formaldehyde and morpholine at 90 °C overnight (Scheme 3) [62]. Compound **5** was converted into the natural nucleoside toyocamycin in five steps.

**Scheme 4:**
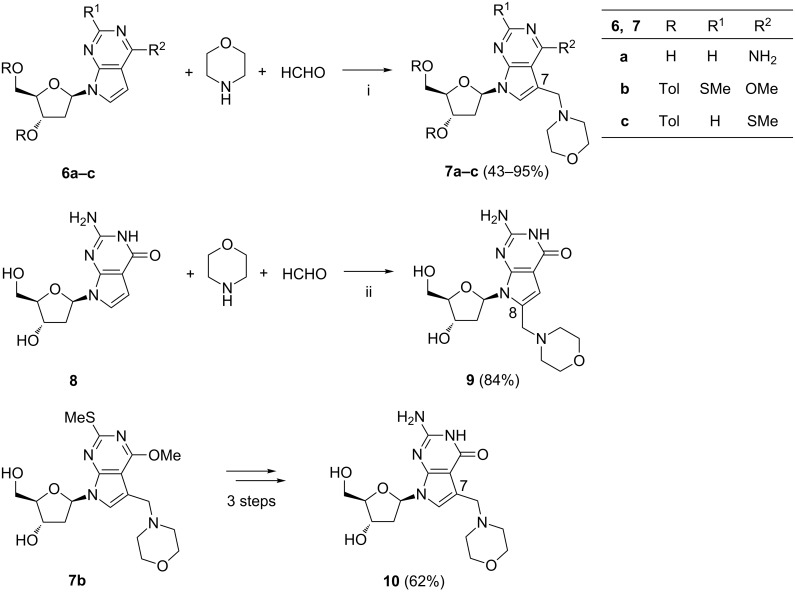
Reagents and reaction conditions: i. AcOH, H_2_O, 60 °C, 12 h-5 d; ii. AcOH, H_2_O, 60 °C, 8 h.

As Seela et al. reported, the reaction conditions developed for the preparation of compound **5** (Scheme 3) were ineffective when applied to 2'-deoxytubercidin **6a** (Scheme 4) [63]. The efficient conversion of compounds **6** to the 7-(morpholinomethyl) derivatives **7** required the use of acetic acid as a co-solvent. However, in the case of 7-deaza-2'-deoxyguanosine (**8**) the regioselectivity of the reaction changed from the C-7 to the C-8 position of the 7-deazapurine system (Scheme 4). The formation of product **9** could be explained by the influence of the electron-donating properties of the C-2 amino group stabilizing the σ-complex formed during the electrophilic attack at the C-8 carbon atom. Since the attempted acylation of the guanine amino group of **8** did not succeeded in the formation of the C-7-substituted guanosine **10**, the compound was obtained in three steps from derivative **7b** by conventional protecting-group manipulations (Scheme 4).

**Scheme 5:**
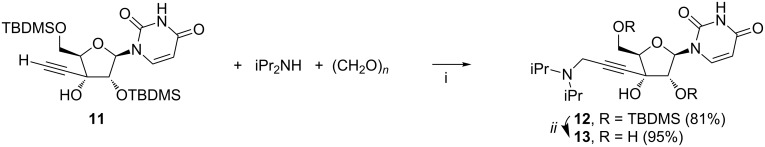
Reagents and reaction conditions: i. CuBr, THF, reflux, 0.5 h; ii. *n*-Bu_4_NF·3H_2_O, THF, rt, 2 h.

The use of 3'-ethynylnucleoside **11** as the alkyne-derived hydrogen active component was described by Dauvergne et al. (Scheme 5) [64]. Treatment of compound **11** with paraformaldehyde and diisopropylamine in the presence of cuprous bromide in refluxing THF afforded the Mannich base **12** in 81% yield. The deprotection of compound **12** with tetrabutylammonium fluoride gave the final product **13**. Compound **13** showed antitumor activity (IC_50_ = 75 µM) against RDM4 tumor cells.

**Scheme 6:**
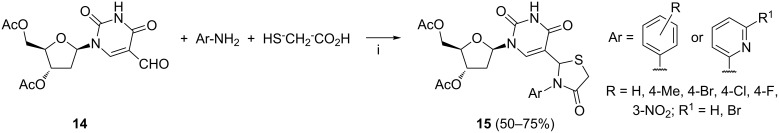
Reagents and reaction conditions: i. [bmim][PF_6_], 80 °C, 5–8 h.

Examples of the Mannich reaction employing a nucleoside as the aldehyde-bearing component are rather limited. Zhang et al. obtained a series of pyrimidine nucleoside-thazolidinone hybrids **15** from 5-formyl-3',5'-di-*O*-acetyl-2'-deoxyuridine (**14**), an arylamine and mercaptoacetic acid (Scheme 6) [65]. The reactions were performed in a ionic liquid ([bmim]PF_6_). Products **15** were obtained in good to moderate yields. Antiparasitic activities of the hybrid compounds **15** were evaluated; some of them showed moderate activities against trypomastigote forms of *Trypanosoma brucei brucei GVR 35* (e.g., IC_50_ = 25 µM for Ar = C_6_H_4_-Cl-4).

**Scheme 7:**
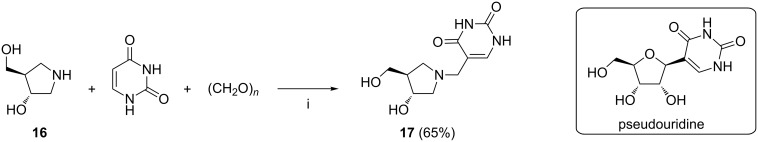
Reagents and reaction conditions: i. EtOH, reflux, 24 h.

**Scheme 8:**
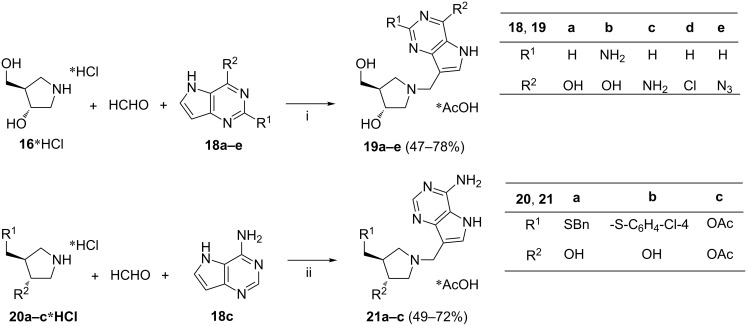
Reagents and reaction conditions: i. NaOAc, H_2_O, 95 °C, 1–16 h; ii. NaOAc, H_2_O, 95 °C, 1 h.

**Scheme 9:**
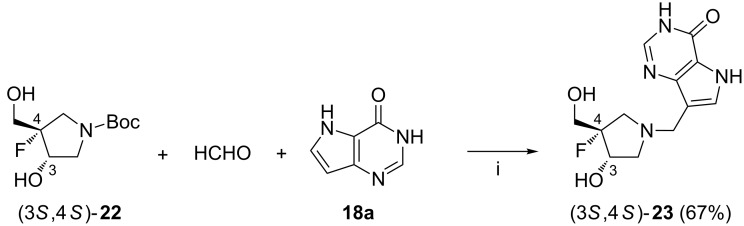
Reagents and reaction conditions: i. a. 37% aq HCl, MeOH; b. NaOAc, 1,4-dioxane, H_2_O, 100 °C, overnight.

The Mannich reaction was also used to construct nucleoside scaffolds from non-nucleoside substrates (Schemes 7–9). Filichev et al. used pyrrolidine **16**, paraformaldehyde and uracil for the preparation of the Mannich base **17**, which is considered as an 1'-aza-analog of pseudouridine (Scheme 7) [66]. Information on application of compound **17** was not given.

By employing pyrrolidine hydrochlorides 16*HCl or 20a–c*HCl (Scheme 8), Evans et al. developed a concise synthesis of 1'-aza-analogs of immucilins, compounds **19** and **21** [67]. The amine hydrochlorides were treated in aq acetate buffer with aq formaldehyde and 9-deazaguanine **18a** or a variety of deazapurines **18b–e**. The acetate buffer was used to generate in situ the free amine **16**, i.e., the Mannich reagent. Reactions leading to products **19** or **21** were conducted for 1 h to 16 h. Among nucleosides **19** and **21**, the 9-deazahypoxanthine-derived compound **19a** (DADMe-Immucilin-H, ulodesine) and the 9-deazaguanine-derived compound **19b** (DADMe-Immucilin-G) were reported to be potent transition state analog inhibitors of human purine nucleoside phosphorylase (PNP). Ulodesine **19a** has completed two phase II clinical trials in 2013 [68–69].

Using the fluorinated pyrrolidine (3*S*,4*S*)-**22** (Scheme 9), Mason et al. obtained azanucleoside (3*S*,4*S*)-**23**, that is an analog of ulodesine **19a** [70]. The two-step procedure leading to compound (3*S*,4*S*)-**23** involved: (i) *N*-Boc-deprotection of (3*S*,4*S*)-**22** with concentrated HCl in methanol, and (ii) treatment of the crude free pyrrolidine with 37% aq formaldehyde and 9-deazahypoxantine **18a** in the presence sodium acetate in dioxane at 100 °C. The compound was prepared on the 10 mg scale in 67% yield. In contrast to its (3*R*,4*R*)-enantiomer (not shown), compound (3*S*,4*S*)-**23** showed inhibitory activity toward human purine nucleoside phosphorylase (PNP) with a slow-onset binding constant *K*_i_* = 0.032 nM. In comparison to ulodesine **19a**, compound (3*S*,4*S*)-**23** exhibited decreased oral availability in mice (0.2 mg/kg dose) and lower duration of action.

**Scheme 10:**
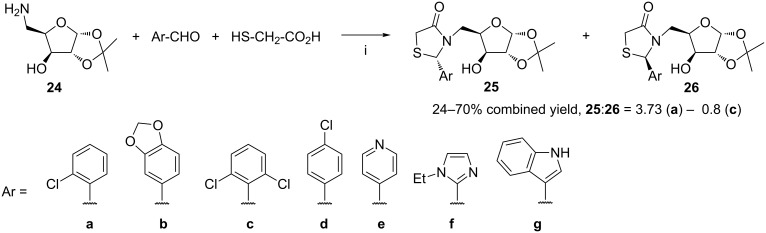
Reagents and reaction conditions: i. DMAP, DCC, MeOH, rt, 1 h.

Compounds **25** and **26**, prepared by Chen et al. [71], can be considered as analogs of reversed nucleosides [72] with the thiazolidin-4-one mimic of a nucleobase (Scheme 10). The compounds were obtained from condensation of aminosugar **24**, arylaldehydes and mercaptoacetic acid in the presence of DMAP and DCC at room temperature. The reaction proceeded with almost no stereoselectivity for the majority of these aldehydes, i.e., two diasteroisomers were isolated in ratios from 0.8 to 1.35. A modest stereoselectivity was observed in the case of 2-chlorobenzaldehyde with the **25a**:**26a** ratio of 3.73. Compounds **25a** and **25b**, in contrast to their isomers **26**, showed moderate activity against human cervical cancer cells at the concentration of 100 µM. Recently, the same group has developed the synthesis of D-glucopyranose-derived counterparts of compounds **25** and **26** [73]. The formation of an intermediate imine from a sugar azide and an aldehyde by Staudinger/aza-Wittig reaction was the key step of the synthesis.

### 2. The Kabachnik–Fields reaction

**Scheme 11:**
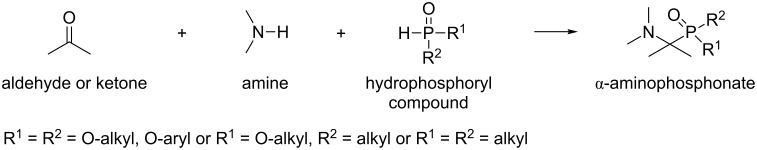
The Kabachnik–Fields reaction.

The Kabachnik–Fields reaction (Scheme 11) proceeds in a three-component system involving a carbonyl compound (aldehyde or ketone), amine, and a hydrophosphoryl compound (mainly alkyl/aryl phosphite) [74–75]. The reaction products, commonly termed as α-aminophoshonates, display properties of industrial and/or medical interest.

**Scheme 12:**
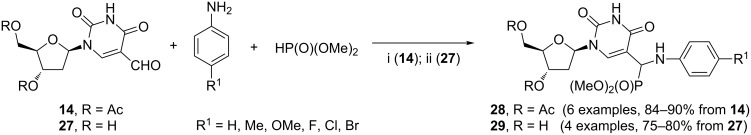
Reagents and reaction conditions: i. 60 °C, 3 h; ii. 80 °C, 2 h.

An example of the application of the Kabachnik–Fields reaction in nucleoside chemistry represents the preparation of α-arylaminophosphonates **28** and **29** by Zhang et al. (Scheme 12) [76]. The reactions between 5-formyl-2'-deoxyuridine **27** (or its 3',5'-di-*O*-acetyl derivative **14**), an aniline and dimethyl phosphite were carried out under solvent-free conditions at 60 °C (for **14**) or at 80 °C (for **27**). Products **28** and **29** were obtained in good to excellent yields as 1:1 diastereoisomeric mixtures arising from the generation of a stereogenic center at the aminophosphonate chain. The mixtures were not separated. Activity of hybrid compounds **28** and **29** against VZV and CMV viruses, as well as against Leishmania donovani promastigotes, was evaluated. Unfortunately, none of them showed any activity up to 250 μM.

### 3. The Ugi reaction

**Scheme 13:**
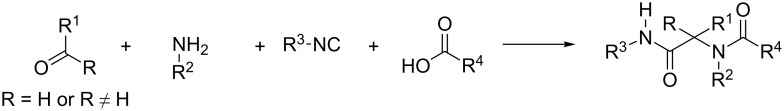
The four-component Ugi reaction.

The Ugi reaction allows for a facile synthesis of a bisamide from a ketone (or an aldehyde), an amine, an isocyanide, and a carboxylic acid (Scheme 13) [77–78]. The Ugi MCRs involving a nucleoside as the substrate bearing the formyl, amino, or isocyano group have been reported.

**Scheme 14:**
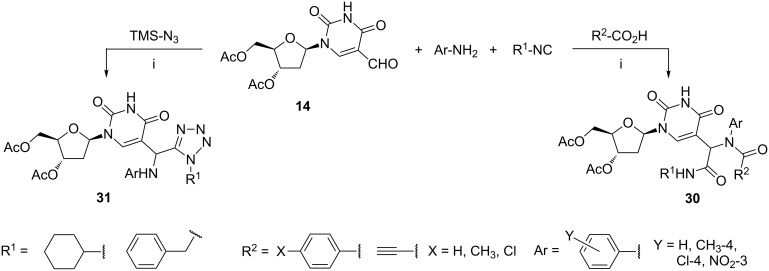
Reagents and reaction conditions: i. MeOH, rt, 2–3 d, yields not given.

The four-component Ugi reaction employing 3',5'-di-*O*-acetyl-5-formyl-2'-deoxyuridine (**14**) as the key substrate afforded nucleosides **30** bearing a *N*-acyl α-amino acid amide moiety at the uracil C-5 carbon atom (Scheme 14) [79]. The variant of the reaction with trimethylsilyl azide (TMS-N_3_) in place of the carboxylic acid gave the tetrazole-substituted nucleosides **31** [79]. Products **30** and **31** were obtained as 1:1 diastereoisomeric mixtures owing to the formation of the new stereogenic center at the amino acid residue. In most cases, the diastereoisomeric mixtures of compounds **30** were separated through column chromatography due to the large differences in the polarity of the diastereoisomers. Anti-leishmanial activity of compounds **30** and **31**, as well as their activity against the vaccinia virus or cowpox virus, were evaluated. Several products **30** displayed moderate anti-leishmanial activity in the range of 12–44 µM.

**Scheme 15:**
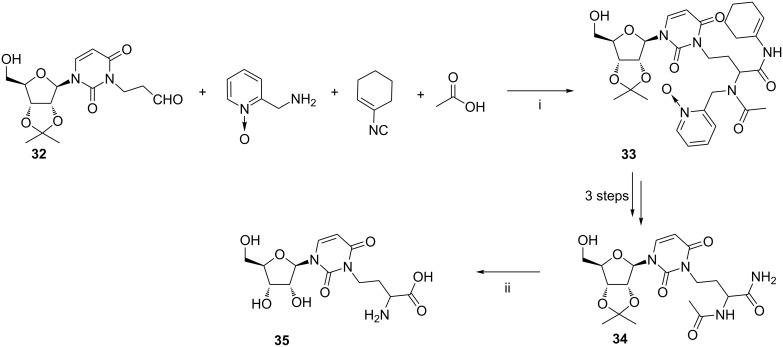
Reagents and reaction conditions: i. MeOH/CH_2_Cl_2_ (1:1), rt, 24 h, yield not given; ii. 6 N aq HCl, 80 °C, 2 h, 80%.

The synthesis of the uridine derivative **35** involving the Ugi condensation as the key step was successfully accomplished by Tsuchida et al. (Scheme 15) [80]. The isopropylidene-protected 3-(2-formylethyl)uridine **32**, 2-(aminomethyl)pyridine 1-oxide, cyclohexenyl isocyanide, and acetic acid were allowed to react under ambient conditions for 24 h to yield the expected product **33**. Further conventional deprotection and acylation steps afforded the intermediate **34**. Upon treatment with 6 N HCl at 80 °C for 2 h the 3-(3-amino-3-carboxypropyl)uridine (**35**) was obtained in 80% yield. While this nucleoside was found in some transfer RNAs, no details of its application were disclosed.

**Scheme 16:**
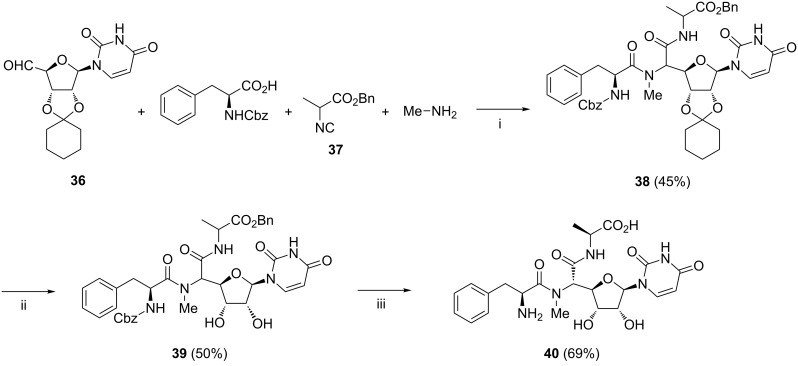
Reagents and reaction conditions: i. MeOH/H_2_O, rt, 26 h; ii. aq AcOH, reflux, 50%; iii. reversed phase HPLC, then MeOH, Pd black, formic acid, 1 h.

Boehm and Kingsbury reported a facile synthesis of *N*-methylated di- and tri-peptide polyoxins by the Ugi reaction (Scheme 16) [81]. The aldehyde **36**, aq methylamine, racemic isonitrile **37**, and (*S*)-*N*-(benzyloxycarbonyl)phenylalanine were combined in MeOH to produce **38** as a mixture of four possible diastereoisomers in a total yield of 45%. The cyclohexylidene protecting group was then removed in refluxing aq AcOH. The resulting diastereoisomers **39** were separated by reversed phase HPLC to yield two pure isomers and the remaining two as an inseparable 1:1 mixture. These were further deprotected by hydrogenolysis under the hydrogen transfer conditions using the Pd black–formic acid system. Only one of the two pure isomers **40** was found to bind to chitin synthase.

**Scheme 17:**
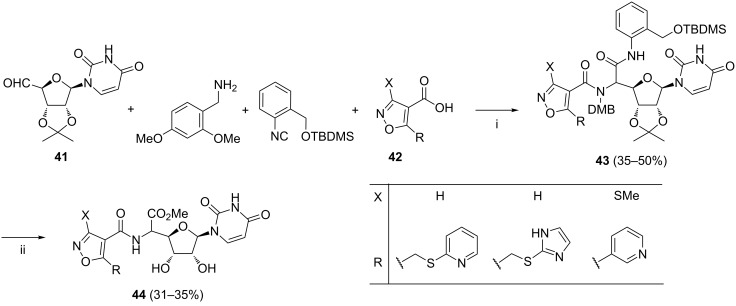
Reagents and reaction conditions: i. MeOH, rt, 24 h; ii. HCl, MeOH, 0 °C to rt, 6 h, then H_2_O, rt, 12 h.

Plant et al. reported another approach to uracil polyoxins via the Ugi reaction [82]. In this work, the desired products **44** were assembled from 2′,3′-protected uridine-5′-aldehyde **41**, 2,4-dimethoxybenzylamine, 2-((*tert*-butyldimethylsilyloxy)methyl)phenylisocyanide, and an isoxazolecarboxylic acid **42** (Scheme 17). Collectively, from three different isoxazolecarboxylic acids **42** three products **43** were obtained (each as ca*.* 1:1 mixture of diastereoisomers). Complete deprotection of **43** was accomplished in methanolic HCl to yield products **44** as mixtures of diastereoisomers.

**Scheme 18:**
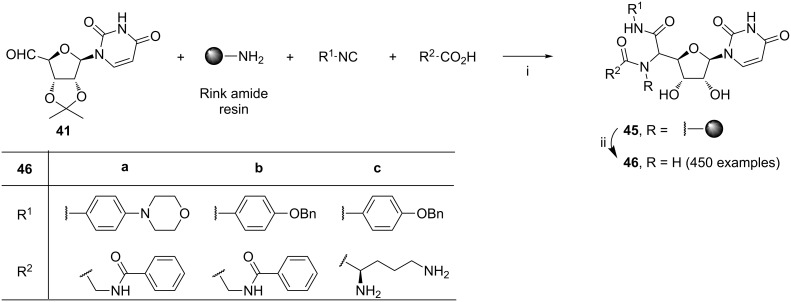
Reagents and reaction conditions: i. DMF/Py/MeOH (1:1:1), rt, 48 h; ii. 10% HCl/MeOH, rt, 30 min.

The Ugi reaction has been often used in solid-phase synthesis of compound libraries [83]. Suda et al. developed the optimal reaction conditions of the solid-phase Ugi reaction involving Rink amide resin as the amine-bearing component (Scheme 18) [84]. The synthesis of nikkomycin Z analogs **46** aimed in an examination of their ability to inhibit *Candida albicans* chitin synthases. The library consisting of 450 analogs **46** was obtained from: (i) reactions involving nucleoside aldehyde **41**, Rink amide resin, one of 15 isocyanides and one of 59 carboxylic acids per reaction; (ii) treatment of the reaction mixtures with methanolic HCl. Products **46** were obtained as 1:1 mixtures of diastereoisomers. Within the library, 246 compounds showed higher than 50% inhibitory activity against *Candida albicans* chitin synthase 1 at the concentration of 10 µM. Among the most active analogs **46a–c**, compound **46a** showed a comparable activity (IC_50_ = 6.07 µM) as that determined for nikkomycin Z (IC_50_ = 9.49 µM). On the other hand, inhibitory activity of this compound toward *Candida albicans* chitin synthase 2 (IC_50_ = 4.78 µM) was significantly lower than that of nikkomycin Z (IC_50_ = 0.06 µM). The remaining compounds **46** were inactive toward *Candida albicans* chitin synthase 2.

**Scheme 19:**
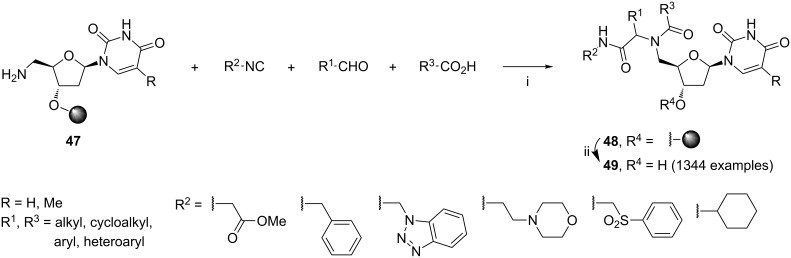
Reagents and reaction conditions (R = CH_3_ or H): i. CH_2_Cl_2_/MeOH (2:1), 35–40 °C, 2 d; ii. HF/pyridine, THF, rt, 2.5 h, then MeOTMS, rt, 3.5 h.

Another approach to the solid-phase synthesis of nucleoside analogs was developed by Sun and Lee (Scheme 19) [85]. The library of 1344 compounds **49** was obtained for antibacterial screening. In this report, 5'-azidothymidine or 5'-azido-2'-deoxyuridine was linked to a polystyrene butyldiethylsilane resin and subsequently reduced to the polymer-supported thymidinyl (R = CH_3_) or 2′-deoxyuridinyl (R = H) aminonucleoside **47**. The library synthesis was executed in 96-well plates, with one of the two amines **47**, 12 carboxylic acids, 8 aldehydes, and an isocyanide per plate. The products **49** were cleaved from the support with HF/pyridine in THF. As expected, the Ugi products **49** were obtained as ca. 1:1 mixtures of diasteroisomers (based on HPLC and ^1^H NMR analysis). Members of this library were claimed to show promising biological activity, however details were not given.

**Scheme 20:**
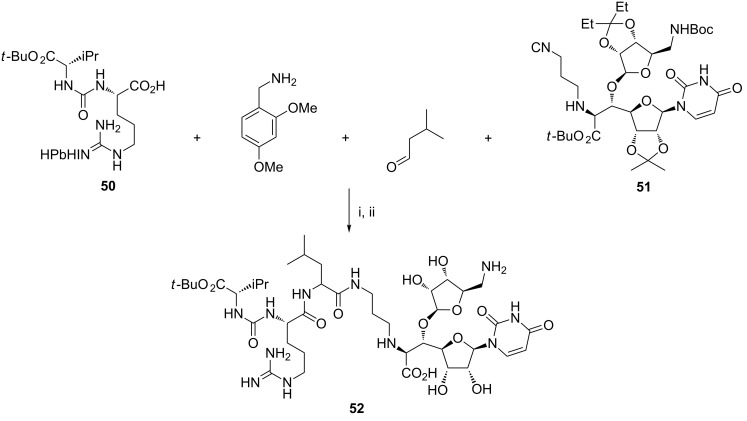
Reagents and reaction conditions: i. MeOH, 76%; ii. 80% aq TFA, 100%.

Muraymycins (MRYs) are a class of naturally occurring nucleoside-lipopeptide antibiotics with excellent antibacterial activity. Matsuda and coworkers envisaged that MRYs complex molecular structure could be efficiently assembled with the help of the Ugi reaction as the key step at the end of their synthesis. This approach was first exercised with a ring-opened muraymycin D2 analogue (Scheme 20) [86]. The reaction of carboxylic acid **50**, 2,4-dimethoxybenzylamine, isovaleraldehyde, and isonitrile-substituted nucleoside **51** in methanol yielded the desired product as a 1:1 mixture of diastereoisomers, which were fully deprotected using aq TFA to furnish the muraymycin analogue **52**.

**Scheme 21:**
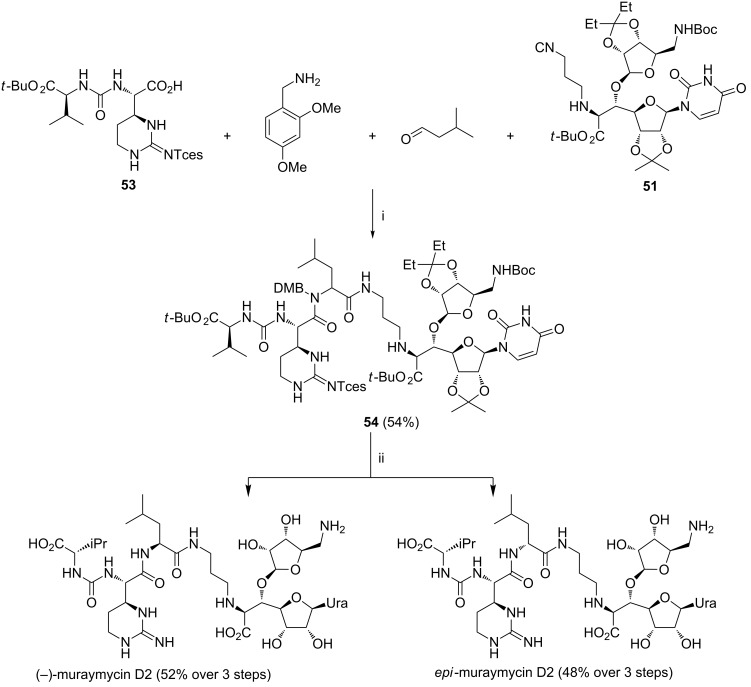
Reagents and reaction conditions: i. EtOH, rt, 72 h; ii. Zn, aq NaH_2_PO_4_, THF, rt, 1 week; then 80% aq TFA, rt, 8 h; then HPLC separation.

This successful route to the MRYs was then applied in the total synthesis of muraymycin D2 and its epimer (Scheme 21) [87]. After completion of the synthesis of the urea dipeptide **53** bearing the cyclic moiety found in muraymycin D2, the four-component condensation was performed similarly as in [86] to yield the protected product **54** as a 1:1 diastereomeric mixture. Functional group manipulation and HPLC separation completed the total synthesis. This approach was further developed in the synthesis of a number of MRY analogues in the following paper from the same research group [88].

**Scheme 22:**
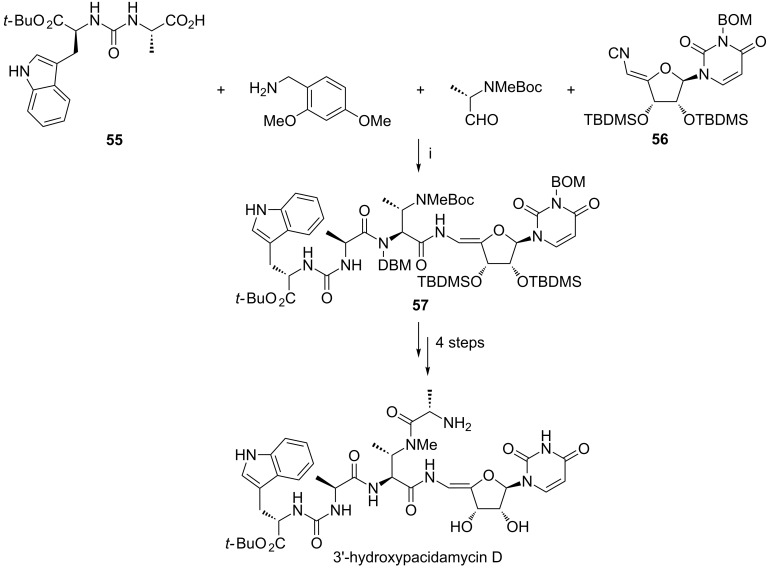
Reagents and reaction conditions: i. EtOH, rt, 48 h, then silica gel chromatography, 33% for **57** (30% for *epi*-**57**).

More recently, the Ugi reaction was applied at a late stage of the synthesis of 3′-hydroxypacidamycin D (Scheme 22) [89]. The urea dipeptide **55**, 2,4-dimethoxybenzylamine, the protected (*S*)-2-(methylamino)propanal, and isonitrile **56** were simply combined in ethanol at ambient temperature for 48 h. The expected compound **57** and its epimer were obtained in reasonable yields, and were separated by column chromatography. The syntheses of 3′-hydroxypacidamycin D and its epimer were then accomplished in four steps from intermediates **57** or *epi*-**57**, including selective deprotection of the *N*-methyl-Boc group, coupling with *N*-Boc-L-alanine, and global deprotection. This strategy was also applicable to the synthesis of a considerable number of pacidamycin analogues.

### 4. The multicomponent domino reactions initiated by the Knoevenagel condensation

The Knoevenagel condensation can be considered as one of the most useful tools for the formation of C=C double bonds. The condensation products, i.e., electron-deficient alkenes, readily act in subsequent reactions as Michael acceptors, Diels–Alder (hetero)dienes or dienophiles, or dipolarophiles. Multicomponent domino reactions initiated by the Knoevenagel condensation are a valuable tool for the construction of many compounds with complex molecular structures [90].

**Scheme 23:**
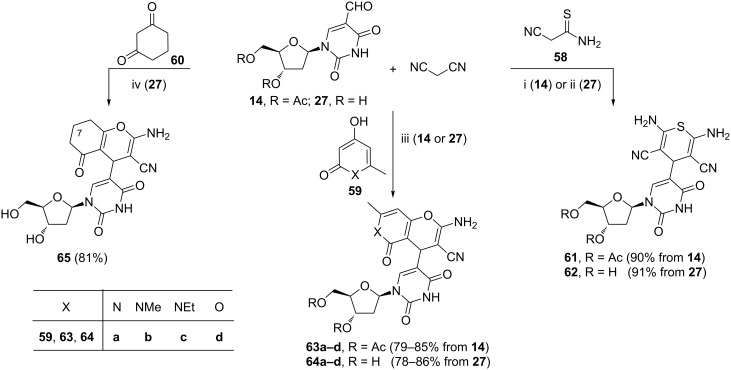
Reagents and reaction conditions: i. [bmim]BF_4_, 80 °C, 4 h; ii. [bmim]BF_4_, 80 °C, 3 h; iii. [bmim]BF_4_, 80 °C, 2–4 h; iv. EtOH, 50 °C, overnight.

**Scheme 24:**
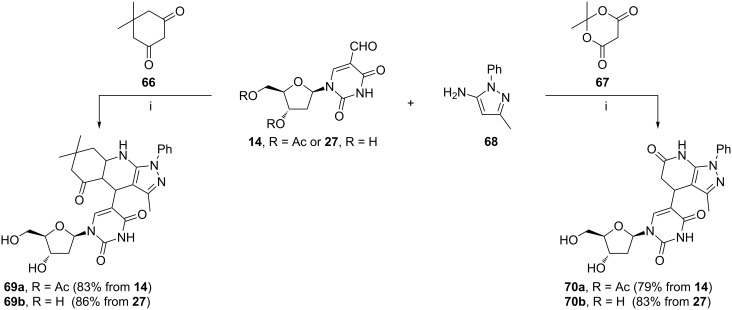
Reagents and reaction conditions: i. [bmim]BF_4_, 80 °C.

The syntheses shown in Scheme 23 and Scheme 24 represent examples of the Knoevenagel condensation-initiated domino reactions where the nucleoside aldehyde (i.e., 5-formyl-3',5'-di-*O*-acetyl-2'-deoxyuridine (**14**) or 5-formyl-2'-deoxyuridine (**27**)) acted as the Knoevenagel acceptor. Compounds **61** to **65** were prepared by the three-component process involving the Knoevenagel condensation, the Michael addition and the Thorpe–Ziegler heterocyclization (Scheme 23). Malonitrile acted as the Knoevenagel donor in all cases. The subsequent Michael addition steps involved: cyanothioacetamide [91], 4-hydroxy-6-methylpyridin-2(1*H*)-one (**59a**) (X = NH) [92], the *N*-methyl-4-hydroxy-6-methylpyridin-2(1*H*)-one (**59b**) (X = NMe) [92], *N*-ethyl-4-hydroxy-6-methylpyridin-2(1*H*)-one (**59c**) (X = NEt) [92], 4-hydroxy-6-methyl-2*H*-pyran-2-one (**59d**) (X = O) [92], or cyclohexane-1,3-dione (**60**) [93]. The syntheses of derivatives **61** to **64** represent a successful application of [bmim]BF_4_ as a solvent [91–92]. The use of the ionic liquid allowed to shorten the reaction time and resulted in much higher yields of the final compounds than those obtained from the reactions performed in conventional organic solvents [91]. Studies on recovery and reuse of [bmim]BF_4_ revealed that this solvent, when used in the fifth reaction cycle, still produced the target product in a good yield [92]. Biological activities of hybrids **63**, **64** and **65** were evaluated [91–92]. Among them, hybrid **63a** exhibited anti-leishmanian activity (IC_50_ = 10.6 ± 1.3 µM) [92]. The SAR study showed that the acetylation of the furanose hydroxy groups resulted in a dramatic decrease in anti-leishmanian activity from 10.6 ± 1.3 µM (**63a**) to 139 µM (**64b**). Compound **65** was active against the cowpox virus in human foreskin fibroblast cells (EC_50_ = 2.0 ± 0.3 µM) [93] and showed anti-leishmanian activity (IC_50_ = 1.4 ± 0.1 µM) [42]. Anti-leishmanian activities of the 7-substituted derivatives of compound **65** were also given [42]. Details concerning the preparation of those compounds were not given.

The three-component synthesis of compounds **69** and **70** developed by Zhang et al. involved the Knoevenagel condensation, the Michael addition, and the *N*-nucleophilic cyclization (Scheme 24) [94]. Whereas 5,5-dimethylcyclohexane-1,3-dione (**66**) or Meldrum’s acid (**67**) acted as the Knoevenagel donor, 3-methyl-1-phenyl-1*H*-pyrazol-5-amine (**68**) played the role of the Michael donor in these reactions. The yields of products **69b** and **70b** derived from 5-formyl-2'-deoxyuridine (**27**) were slightly higher than yields of derivatives **69a** and **70a** obtained from the *O*-acetylated nucleoside **14**.

**Scheme 25:**
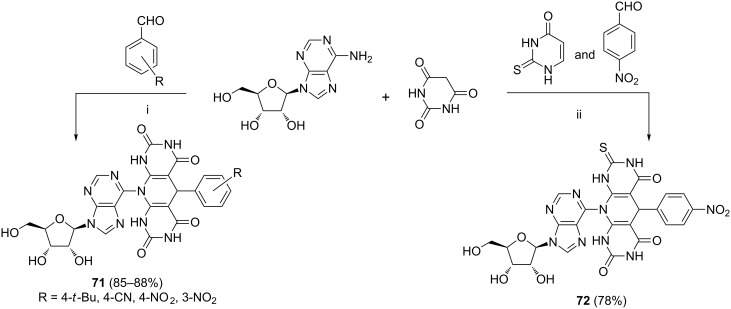
Reagents and reaction conditions: i. H_3_PW_12_O_40_ (2 mol %), EtOH, 50 °C, 2–15 h; ii. H_3_PW_12_O_40_ (2 mol %), EtOH, 50 °C, 8 h.

The syntheses of hybrids **71** [95] and **72** [96] represent examples of the Knoevenagel-initiated domino reactions where the purine nucleoside (i.e., adenosine) was modified (Scheme 25). Tungstophosphoric acid (H_3_PW_12_O_40_) was employed as a catalyst (2 mol %). Hybrids **71** originated from the pseudo-four component cascade employing two equivalents of barbituric acid. The authors demonstrated that the method was applicable with both electron-poor and electron-rich aldehydes. The four-component variant of the reaction employing 2-thiouracil led to compound **72** with a slightly lower yield than those obtained from pseudo-four component cascade leading to compounds **71**.

### 5. The Biginelli reaction

**Scheme 26:**
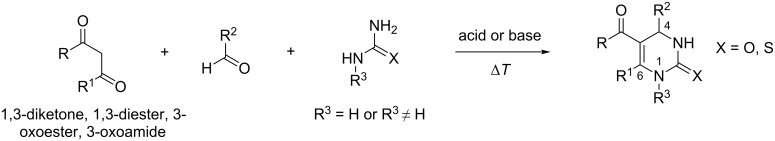
General scheme of the Biginelli reaction.

The Biginelli reaction (Scheme 26) consists in the three-component condensation of a 1,3-dicarbonyl compound, an aldehyde, and a nitrogen component, i.e., urea (X = O, R^3^ = H) or thiourea (X = S, R^3^ = H) [29]. The use of *N*-substituted derivatives of urea or thiourea (R^3^ ≠ H) has also been reported. Recently numerous advances in the asymmetric Biginelli reaction have been reviewed [97]. The reaction has been employed in the synthesis of C-nucleosides with 3,4-dihydropyrimidin-2(1*H*)-one or 3,4-dihydropyrimidin-2(1*H*)-thione as the nucleobase mimic. Up to date, depending on the role of the carbohydrate component in the reaction, C-nucleosides bearing the carbohydrate moiety at the position of N-1, C-4 or C-6 of the nucleobase mimic were synthesized.

**Scheme 27:**
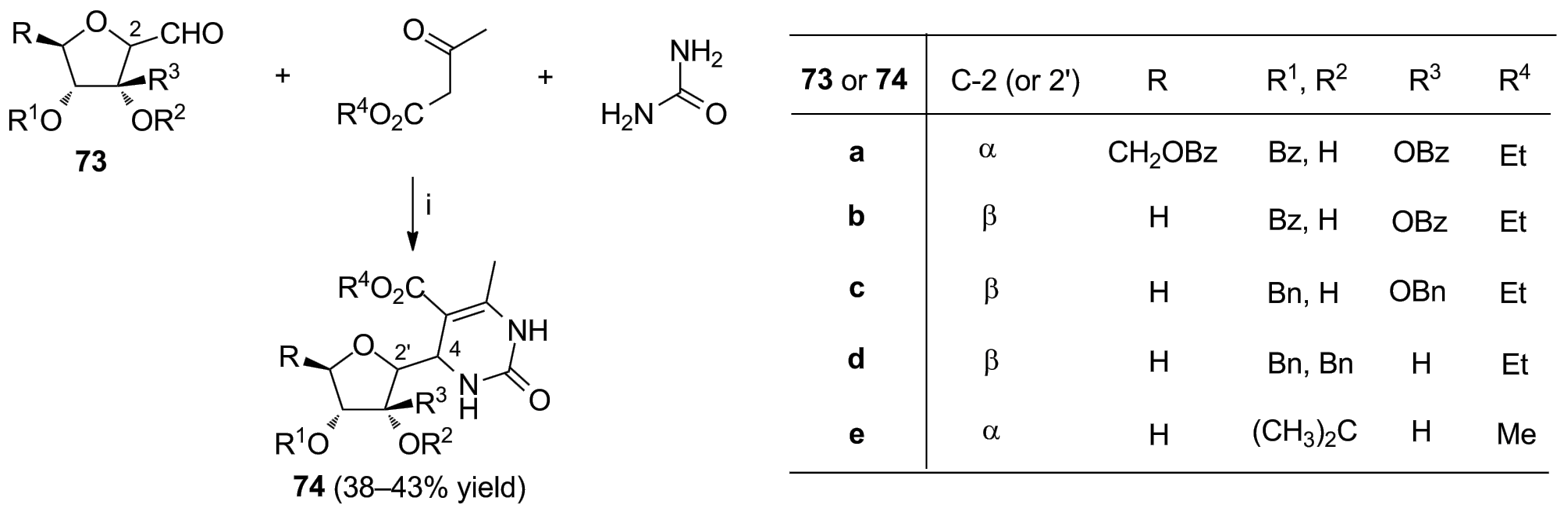
Reagents and reaction conditions: i. EtOH, reflux.

Starting from sugar aldehyde substrates **73**, Molina et al. synthesized a series of compounds **74** bearing the carbohydrate moiety at the C-4 carbon atom of the 3,4-dihydropyrimidin-2(1*H*)-one system (Scheme 27) [98–99]. Attempts to replace the aldehyde **73a** with its 3,4,6-hydroxylated counterpart failed to give the expected product [99].

**Scheme 28:**
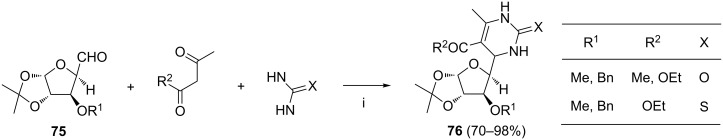
Reagents and reaction conditions: i. Bu_4_N^+^HSO_4_^−^, diethylene glycol, 120 °C, 1.5–3 h.

Dwivedi et al. showed that the isopropylidene-protected sugars **75** reacted efficiently with urea (or thiourea) and 1,3-dicarbonyl compounds in diethylene glycol in the presence of tetrabutylammonium hydrogen sulfate as both an acid and a phase-transfer catalyst (Scheme 28) [100]. As the authors suggested, the formation of intermediate *N*-acyliminium ion from aldehyde **75** and (thio)urea was the key step of the reaction. Protonation of aldehyde **75** by tetrabutylammonium hydrogen sulfate facilitated the reaction. Galactose-6'-aldehyde counterparts of the urea-derived compounds **76** (X = O) were also prepared by this method.

**Scheme 29:**
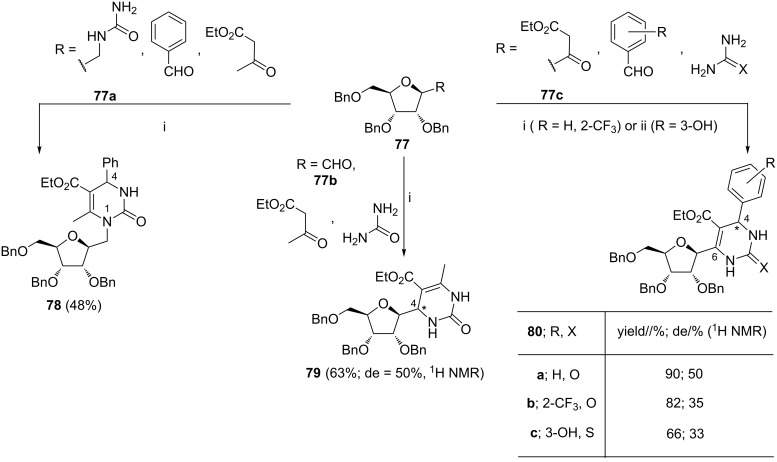
Reagents and reaction conditions: i. BF_3_·Et_2_O, CuCl, AcOH, THF, 65 °C, 24 h; ii. Yb(OTf)_3_, THF, reflux, 20 h.

The Dondoni group developed Lewis acid-promoted reactions employing the sugar derivatives **77** acting as: the component bearing the urea function (**77a**), the aldehyde function (**77b**), or the β-ketoester function (**77c**) (Scheme 29) [101–102]. In contrast to the N-1-substituted homo-C-nucleosides **78**, the C-4 or C-6-substituted C-nucleosides (i.e., compounds **79** or **80**, respectively) were obtained with the diastereoisomeric excess varied from 33% to 50%. The diastereoisomers were separated and their absolute configuration was determined using X-ray crystallography and circular dichroism spectroscopy. The stereochemical outcome of the synthesis of compounds **79** and **80** was suggested to result from some internal asymmetric induction of the chiral residue of the sugar aldehyde **77b** or the sugar β-ketoester **77c**, respectively. The debenzylated forms of *C*-nucleosides **78**, **79** and **80** (as single diastereoisomers) were evaluated in vitro and in vivo as antimitotic agents [41]. They appeared to be less active than the reference (4*S*)-monastrol. Pyranose-derived nucleoside analogs were also prepared by these methods [101–102].

**Scheme 30:**
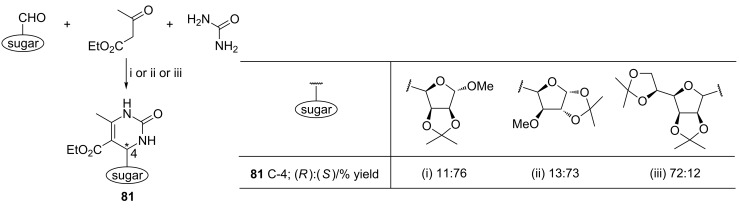
Reagents and reaction conditions: TCT (10 mol %), rt: i. 100 min; ii. 150 min; iii. 140 min.

Sharma et al. used 2,4,6-trichloro[1,3,5]triazine (TCT) as the source of hydrogen chloride to promote the reactions leading to C-4-substituted C-nucleosides **81** with the high (ca. 7:1) diastereoisomeric ratio (Scheme 30) [103]. The products were isolated as single diastereoisomers. Since the reactions conducted in the presence of molecular sieves (4Å) were unsuccessful, the authors suggested that traces of moisture present in the reaction system played the key role in the release of hydrogen chloride from TCT. A pyranose-derived nucleoside analog was also prepared by this method.

**Scheme 31:**
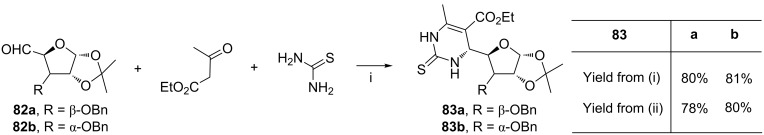
Reagents and reaction conditions: i. EtOH, microwave irradiation (300 W), 10 min; ii. EtOH, 75 °C, 5 h and for 72 h, rt.

Very recently, Figueiredo et al. synthesized C-nucleosides **83** with the C-4 substituted 3,4-dihydropyrimidin-2(1*H*)-thione as a nucleobase (Scheme 31) [104]. The products were obtained as the C-4-(*R*) single diastereoisomers. The use of microwave irradiation allowed the authors to perform these reactions with ten times smaller volume of the solvent than that employed in the reactions carried out under conventional heating conditions. Compound **83b** showed promising activity against acetylcholinesterase at a concentration of 100 µmol/L.

### 6. The Hantzsch reaction

**Scheme 32:**
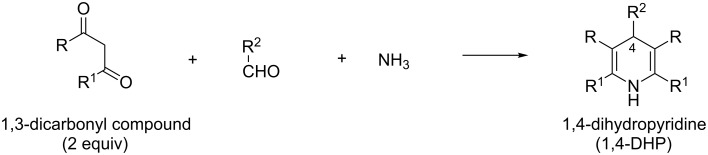
The Hantzsch reaction.

The classical Hantzsch reaction provides 1,4-dihydropyrimidines (1,4-DHPs) from 1,3-dicarbonyl compounds, aldehydes and ammonia (Scheme 32) [19]. The reaction has attracted a considerable attention because of the therapeutic usefulness of drugs featuring the 1,4-DHP scaffold, i.e., nifedipine and olanzapine [105]. The preparation of unsymmetrical 1,4-DHPs by the Hantzsch reaction involving two different β-ketoesters has been reported [106]. The literature survey revealed that the Hantzsch reaction served as a tool for the preparation of C-nucleosides with the C-4-substituted 1,4-DHP moiety as a nucleobase (R^2^ = sugar).

**Scheme 33:**
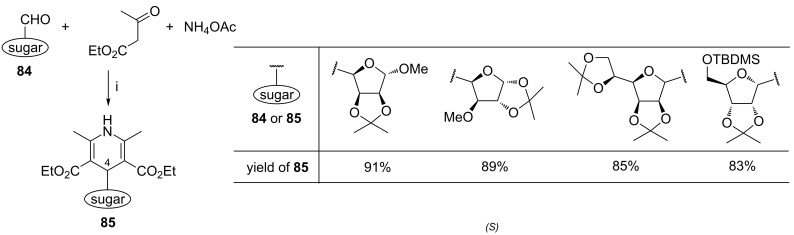
Reagents and reaction conditions: TCT (10 mol %), rt, 80–150 min.

The Hantzsch reaction involving the sugar-derived aldehydes **84**, ethyl acetoacetate and ammonium acetate was applied by Sharma et al. in the synthesis of nucleoside analogs **85**, bearing the 1,4-DHP nucleobase at the C-4- or C-1 carbon atom of the sugar (Scheme 33) [107]. Analogously to the previously reported Biginelli reaction [103], compounds **85** were obtained in high yields under the TCT-catalysis conditions. A pyranose-derived nucleoside analog was also prepared by this method.

**Scheme 34:**
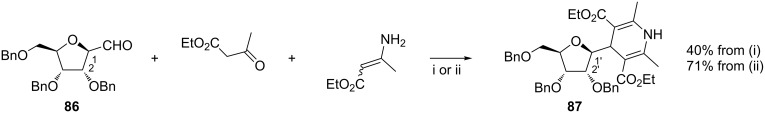
Reagents and reaction conditions: i. Yb(OTf)_3_, THF, 90 °C, 12 h; ii. 4 Å molecular sieves, EtOH, 90 °C, 48 h.

Using compound **87** as an example (Scheme 34), the Dondoni group demonstrated that the C-nucleosides with the C-4-substituted 1,4-DHP nucleobase can be efficiently obtained from the three-component reaction between the sugar aldehyde **86**, ethyl acetoacetate, and ethyl 3-aminocrotonate [108–109]. The course of the reaction in the presence of various additives was examined in detail. The best results were obtained in the presence of 4 Å molecular sieves. The analysis of the reaction products showed that ytterbium triflate induced partial 1,2-elimination of benzyl alcohol from the ribosyl residue of the starting aldehyde **86**, consequently leading to the 1',2'-didehydro-derivative of the target product **87**. Pyranose-derived nucleoside analogs were also prepared by this method [108–109].

**Scheme 35:**
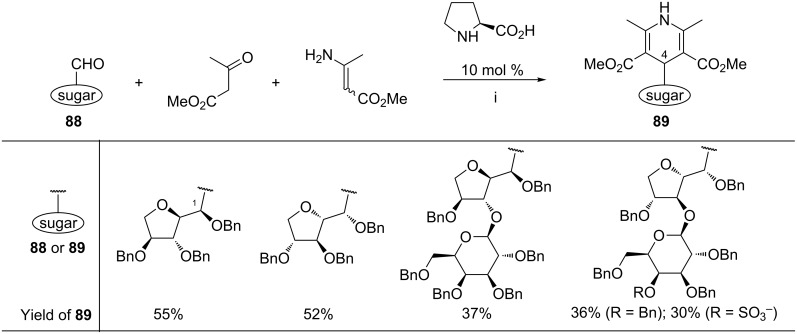
Reagents and reaction conditions: i. MeOH, 50 °C, 48 h.

This approach involving a sugar aldehyde, 3-oxoester, and an ester of 3-aminocrotonic acid was then extended by the Dondoni group to 2,5-deoxyhexose-derived aldehydes **88** (Scheme 35) [110]. The best results were obtained when the reaction was performed with an excess (1.5 equiv) of methyl acetoacetate and methyl 3-aminocrotonate under L-proline-catalyzed conditions. In contrast to other catalysts tested (ytterbium triflate, D-proline, (*S*)-5-(pyrrolidin-2-yl)-1*H*-tetrazole, or (*S*)-1-(pyrrolidin-2-ylmethyl)pyrrolidine/TFA system), the catalytic effect of L-proline resulted in an increase in the reaction yield. Moreover, epimerization on the C-1 carbon atom of the starting aldehyde **88** was also suppressed. The latter effect was attributed to the preferential activation of methyl 3-aminocrotonate by L-proline via the corresponding enamine as compared to the activation of the sugar aldehyde.

**Scheme 36:**
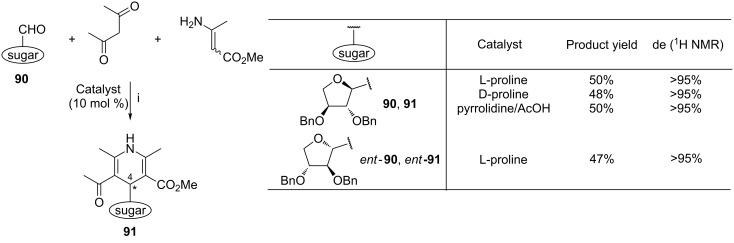
Reagents and reaction conditions: i. MeOH, 25 °C, 5 d.

The preliminary studies of the Dondoni group on the synthesis of C-nucleosides bearing the unsymmetrical 1,4-dihydropyridine nucleobase showed that the internal asymmetric induction by the sugar moiety played a crucial role in the formation of compounds **91** (Scheme 36) [110]. Regardless of the catalyst used, aldehyde **90** gave product **91** with a very high diastereomeric excess. Analogously to the reaction performed with aldehyde **90** in the presence of L-proline, aldehyde *ent*-**90** gave compound *ent*-**91** with the same diastereomeric excess under the same conditions. The absolute configuration of the C-4 carbon atom of compound **91** or *ent-***91** was not determined.

**Scheme 37:**
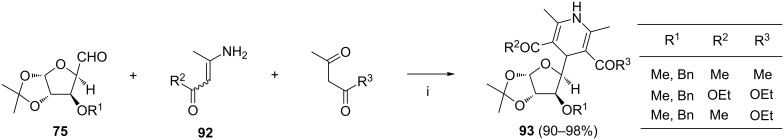
Bu_4_N^+^HSO_4_^−^, diethylene glycol, 80 °C, 1–2 h.

The approach involving an enamine (i.e., compound **92**) as one of the reaction components was also used by Tewari et al. for the preparation of C-nucleosides **93** (Scheme 37) [111]. The reactions were carried out in the presence of tetrabutylammonium hydrogen sulfate as a phase-transfer catalyst. The yield of products **93** varied from 90% to 98%. As the authors suggested on the basis of comparative experiments performed without the catalyst, tetrabutylammonium hydrogen sulfate facilitated dehydration and cyclization steps of the reaction owing to its acidic properties. The reaction variant involving the corresponding sugar aldehyde **75**, 4-aminopent-3-en-2-one and ethyl 3-oxobutanoate allowed to obtain unsymmetrical products **93**. Galactose-6'-aldehyde-derived counterparts of the symmetrical nucleosides **93** were also prepared by this method.

### 7. The carbopalladation of dienes

**Scheme 38:**
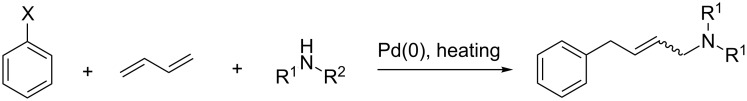
The three-component carbopalladation of dienes on the example of buta-1,3-diene.

A reaction of an aryl halide, an unsaturated alkene (diene, allene), and an amine catalyzed by Pd(0) species, referred to as carbopalladation of dienes, results in the three-component assembly of an unsaturated amine (Scheme 38) [112].

**Scheme 39:**
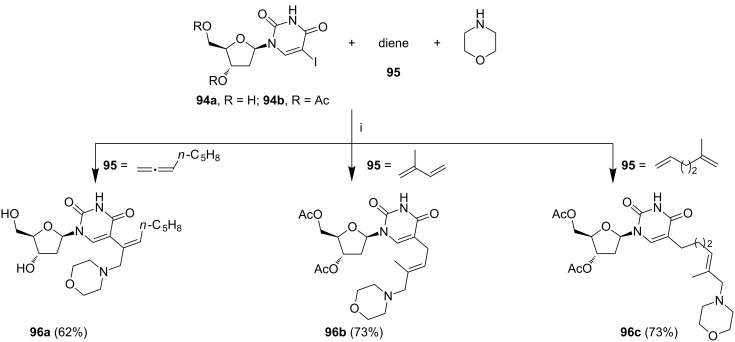
Reagents and reaction conditions: i. 5 mol % Pd(dba)_2_, Bu_4_NCl, ZnCl_2_, acetonitrile or DMSO, 80 °C or 100 °C, 1–2 days.

The palladium-catalyzed reactions of 5-iodopyrimidines, various acyclic or cyclic dienes, and amines were optimized by Larock et al. [113]. Thus, coupling of 5-iodo-2'-deoxyuridine (**94a**) or 3',5'-di-*O*-acetyl-5-iodo-2'-deoxyuridine (**94b**) with 1,2-, 1,3- or 1,ω-dienes **95**, and morpholine afforded a considerable variety of the corresponding 5-(alkylallylamino)-2'-deoxyuridines **96** (Scheme 39, selected examples are shown). After an extensive search for optimal reaction conditions, the authors found that the best yields could be achieved in the presence of zinc salts, in particular with secondary amines. In some cases, protection of the hydroxy groups in **94a** was also necessary. The reactions between 3',5'-di-*O*-acetyl-5-iodo-2'-deoxyuridine (**94b**), long-chain 1,ω-dienes (e.g., deca-1,9-diene or tetradeca-1,13-diene) and morpholine afforded products as mixtures of regioisomers resulting from the addition of the nucleoside moiety to the C-1 or C-2 carbon atom of the C=C double bond.

**Scheme 40:**
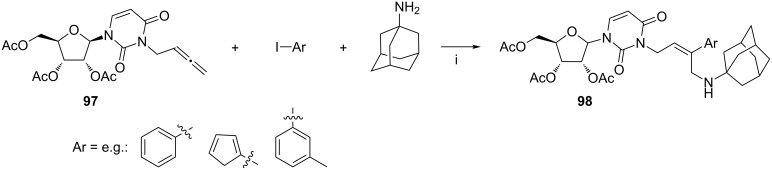
Reagents and reaction conditions: i. 2.5 mol % Pd_2_(dba)_3_, tris(2-furyl)phosphine, K_2_CO_3_, MeCN or DMF, 80 °C, 3–24 h, 77–99%.

The three-component reactions of nucleoside-derived (uridine or thymidine) allenes **97**, a range of aryl iodides, and 1-adamantylamine was accomplished smoothly under the palladium-catalyzed conditions (Scheme 40, the uridine example is shown) [114]. The coupling products **98** were obtained as (*Z*)-stereoisomers for studies related to the drug discovery against the hepatitis C virus.

**Scheme 41:**
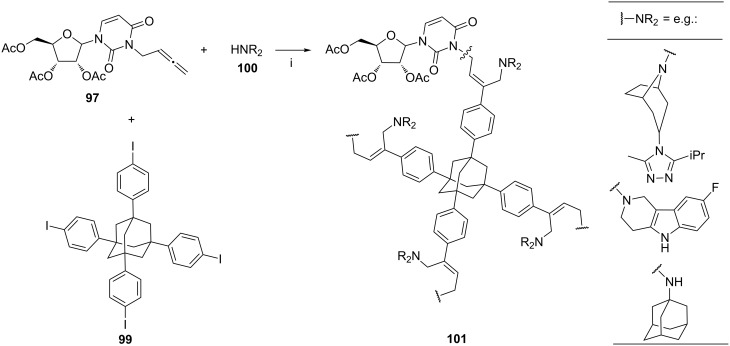
Reagents and reaction conditions: i. 2.5 mol % Pd_2_(dba)_3_, tris(2-furyl)phosphine, K_2_CO_3_, MeCN or DMF, 80 °C, 3–24 h, 45–87%.

The methodology shown in Scheme 40 [114] was further elaborated on reactions of polyfunctional iodide **99** with four equivalents of nucleoside-derived allenes **97** (the uridine example shown), and a number of amines **100** (four equivalents, Scheme 41). The polyfunctional products **101** were obtained with excellent (*Z*)-stereoselectivity. The authors noticed a pronounced relationship between p*K*_a_ of the amine and the isolated yield of the product, i.e., 1-adamantylamine provided the highest yield.

### 8. The Bucherer–Bergs reaction

**Scheme 42:**
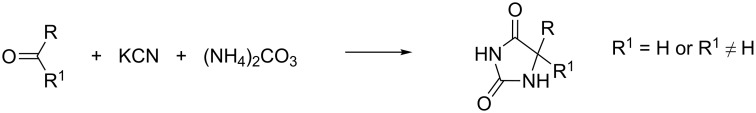
The three-component Bucherer–Bergs reaction.

**Scheme 43:**
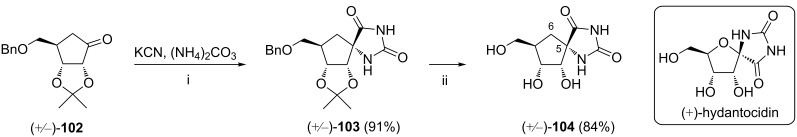
Reagents and reaction conditions: i. MeOH, H_2_O, 70 °C, 4.5 h; ii. (1) H_2_, 5% Pd/C, MeOH, 55 °C, 5 h; (2) Dowex 50 W, MeOH, H_2_O, rt, 4 h.

The three-component Bucherer–Bergs reaction provides 5-mono- or 5,5-disubstituted hydantoins from the condensation of a carbonyl compound with potassium cyanide and ammonium carbonate (Scheme 42) [115]. The chemistry of hydantoins attracted a considerable attention because of their importance in medicine and industry [116–117]. N-Nucleoside analogs with (thio)hydatantoin scaffold as a nucleobase mimic were also extensively investigated [118].

(+)-Hydantocidin (Scheme 43), isolated from *Streptomyces hygroscopicus*, is a unique nucleoside with a spirohydantoin ring at the anomeric carbon atom of D-ribofuranose. (+)-Hydantocidin has been identified as a herbicidal or a plant growth regulatory agent [119]. Using the Bucherer–Bergs reaction, Sano and Sugai accomplished the synthesis of a racemic 5-*epi*-6-carba-analog of (+)-hydantocidin (Scheme 43, compound (+/−)-**104**) [120]. The key step of the synthesis involved condensation of the racemic ketone (+/−)-**102** with potassium cyanide and ammonium carbonate in aq methanol at 70 °C. The 5-*epi* configuration of compound (+/−)-**103** was confirmed by NMR spectroscopy. In contrast to the (+/−)-6-carba-analog of (+)-hydantocidin, compound (+/−)-**103** was devoided of herbicidal activity at 1000 ppm concentration.

### 9. Miscellaneous reactions

**Scheme 44:**
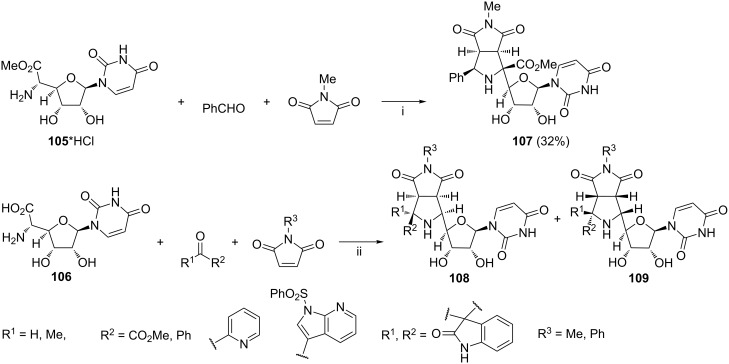
Reagents and reaction conditions: i. pyridine, MgSO_4_, 100 °C, 28 h, N_2_; ii. DMF, 70–90 °C, 22–30 h, N_2_, the cumulative yield of **108**/**109** from 55% to 96%.

Dondas et al. reported the synthesis of a derivative of compound **107** bearing the pyrrolo[3,4-*c*]pyrrole skeleton at the furanose C-4' position from uracil polyoxin C hydrochloride 105*HCl (Scheme 44) [121]. The reaction cascade involved thermal formation of the corresponding azomethine ylide from substrate 105*HCl and benzaldehyde, followed by 1,3-dipolar cycloaddition of the ylide to *N*-methylmaleimide. The formation of compound **107** as the only product was rationalized using semi-empirical calculations. In the same contribution, the cascade reactions starting from uracil polyoxin C **106** were described (Scheme 44). Decarboxylative formation of azomethine ylides from **106** and an aldehyde (or ketone), followed by reaction of the ylide with maleimide afforded mixtures of cycloadducts **108** and **109** in molar ratios varied from 1:1 to 12:1. Compounds **108** were inactive against *Aspergillus fumigatus* or *Candida albicans* at concentration of 125 µg/mL.

**Scheme 45:**
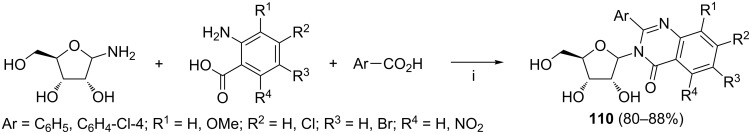
Reagents and reaction conditions: i. Montmorillonite K-10 clay, microwave irradiation (600 W), 6–10 min (solvent-free conditions).

Nucleoside analogs **110** with the 3,4-dihydroquinazoline-derived nucleobase were obtained by Siddiqui et al. under microwave irradiation conditions with the substrates adsorbed onto Montmorillonite K-10 clay (Scheme 45) [122]. The formation of compounds **110** proceeded via: (a) N-acylation of aminosugar by the anthranilic acid derivative, and (b) N-acylation of the resulting amide at the aromatic amino group by benzoic acid (or 4-chlorobenzoic acid), followed by cyclization of the resulting diamide intermediate. After completion of the irradiation, products **110** were extracted with dichloromethane from the clay and crystallized from ethanol.

**Scheme 46:**
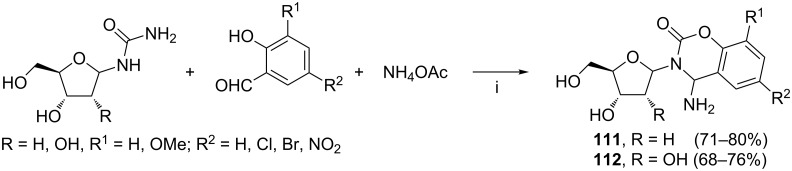
Reagents and reaction conditions: i. Montmorillonite K-10 clay, microwave irradiation (560 W), 6–10 min (solvent-free conditions).

The Montmorillonite K-10 clay–microwave irradiation reaction system was also used by Yadav and Rai in the synthesis of nucleoside analogs **111** and **112** bearing novel nucleobase derived from benzo[*e*][1,3]oxazine (Scheme 46) [123]. The developed reactions were much more effective than those examined on other inorganic supports (i.e., silica gel, neutral or basic alumina). The conversion of the sugar urea to the corresponding isocyanate intermediate, accompanied by the loss of ammonia, was postulated to be the key step of the reaction cascade.

**Scheme 47:**
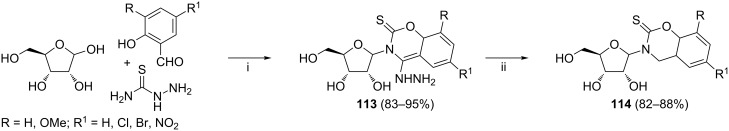
Reagents and reaction conditions: i. CeCl_3_·7H_2_O (20 mol %), NaI (20 mol %), microwave irradiation (90 °C), 6–8 min; ii. CuSO_4_·5H_2_O, Al_2_O_3_, microwave irradiation (90 °C), 3–3.5 min.

Another approach to nucleoside analogs bearing a nucleobase derived from benzo[*e*][1,3]oxazine was developed by Rai and Singh (Scheme 47) [124]. The target compounds **113** and **114** were assembled from the three-component mixture of D-ribose, a derivative of salicylic aldehyde, and thiosemicarbazide under Lewis acid-catalysis and microwave irradiation. In comparison with analogous reactions carried out with mineral support (i.e., Montmorillonite K-10 clay or silica gel), the use of the CeCl_3_/NaI catalyst system for the synthesis of intermediates **113** provided the best results in terms of reaction yield and time. The next step leading to final products **114**, i.e., the reductive dehydrazination of compounds **113** with alumina-supported copper(II) sulfate was conducted under solvent-free microwave irradiation conditions. Products **114** were isolated by crystallization in yields exceeding 80%.

**Scheme 48:**
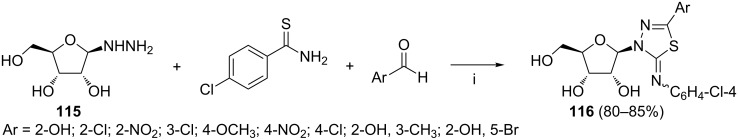
Reagents and reaction conditions: i. PhI(OAc)_2_ (3 mol %), microwave irradiation (45 °C), 6–9 min.

Siddiqui et al. developed the method for the preparation of nucleoside analogs **116** with the 1,3,4-thiadiazole-derived nucleobase. This method involved the microwave irradiation-assisted condensation of sugar hydrazine **115**, 4-chlorobenzothioamide and an aromatic aldehyde in the presence of (diacetoxyiodo)benzene (Scheme 48) [125]. The conversion of 4-chlorobenzothioamide to 4-(chlorophenyl)isothiocyanate intermediate by (diacetoxyiodo)benzene was suggested to initiate the reported reaction sequence.

**Scheme 49:**
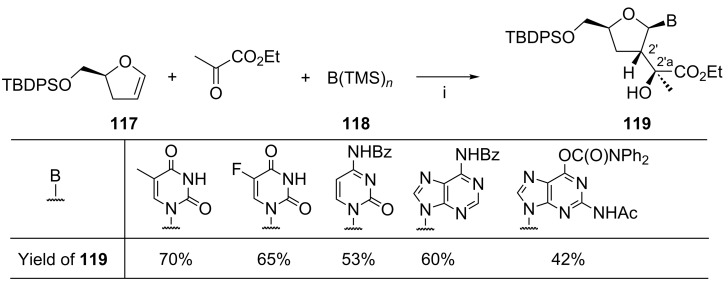
Reagents and reaction conditions: i. **117**, ethyl pyruvate, TiCl_4_, dichloromethane, −78 °C, 1 h; then **118**, −78 °C, 1 h; then 23 °C, 1 h*.*

Ghosh and Kass synthesized nucleosides **119** from 2,3-dihydrofurane **117**, ethyl pyruvate, and the silylated nucleobase **118** (Scheme 49) [126]. This method does not strictly comply with the Ugi’s definition of MCRs because of the sequential addition of the substrates. However, in our opinion the method is worth noting since it represents an interesting extension of the Vorbrüggen N-glycosylation process. Thus, the reaction sequence leading to nucleosides **119** was initiated by the titanium(IV) chloride-promoted alkylation of 2,3-dihydrofurane **117** with ethyl pyruvate at −78 °C (1 hour), followed by the coupling of the resulting oxocarbenium ion with the silylated nucleobase **118**. Compounds **119** were obtained as single diastereoisomers. The similar (not shown) reaction employing the silylated thymine and ethyl glyoxalate gave the corresponding product as 1:1 mixture of isomers at the *C*-2'a carbon atom.

## Conclusion

In this comprehensive review application of multicomponent reactions (MCRs) in nucleoside chemistry has been presented. In recent years, growing interest in the construction of novel nucleoside scaffolds by MCR has been observed. This conclusion is supported by the fact that 23 out of 60 original works cited in this review appeared within the last five years. Up to date, much more efforts were devoted to the preparation of novel nucleoside scaffolds by a structural modification of the parent nucleosides (37 examples) than by their de novo construction from non-nucleoside substrates (23 examples). A majority of the reported modifications of the parent nucleosides concerned their nucleobase moiety (27 examples). However, the number of reports on modifications of the purine nucleobase was limited (4 examples). Among reports on the de novo construction of nucleosides from non-nucleoside substrates, the ones dealing with the construction of a non-natural nucleobase predominated (18 examples). Interestingly, a combinatorial solid-phase approach has not been extensively exploited (2 examples). The findings concerning the syntheses of nucleoside antibiotic analogs or 1'-aza-analogs of immucilins are interesting in view of both organic synthesis and potential applications. The trends of a great research potential in this field could be identified from the presented literature survey. The most recent reports were mainly directed to: (i) the employment of novel reaction techniques, such as microwave irradiation, ionic liquids or inorganic supports, or (ii) the development of novel MCRs leading to nucleoside analogs bearing an unconventional nucleobase. As reports dealing with these issues revealed, a combination of both these trends may result in the preparation of structurally interesting compounds. An intensification of studies on the structure–activity relationship of these compounds would provide valuable data on their potential applications. We hope that continued efforts in this field will result in novel nucleoside drug candidates.

**Table 1:** Abbreviations.

Abbreviation	Term

Ac	acetyl
Ar	aryl
[bmim]BF_4_	1-butyl-3-methylimidazolium tetrafluoroborate
[bmim]PF_6_	1-butyl-3-methylimidazolium hexafluorophosphate
Bn	benzyl
Boc	*tert*-butoxycarbonyl
BOM	benzyloxymethyl
Bu	butyl
Cbz	benzyloxycarbonyl
DCC	*N*,*N*'-dicyclohexylcarbodiimide
DCM	dichloromethane
DMAP	4-(*N*,*N*-dimethylamino)pyridine
DMB	2,4-dimethoxybenzyl
Et	ethyl
EWG	electron withdrawing group
iPr	isopropyl
MCPBA	*m*-chloroperbenzoic acid
Me	methyl
rt	room temperature
SAR	structure activity relationship
TBDMS	*tert*-butyldimethylsilyl
*t*-Bu	*tert*-butyl
Tces	2,2,2-trichloroethoxysulfonyl
TCT	2,4,6-trichloro-1,2,3-triazine
Tf	(trifluoromethyl)sulfonyl
TFA	trifluoroacetic acid
TFP	tris(2-furyl)phosphine
THF	tetrahydrofuran
TMS	trimethylsilyl
Tol	4-methylbenzoyl
Ura	pyrimidine-2,4(1*H*,3*H*)-dion-1-yl
